# Unpacking the Racial Gap: Helicobacter pylori Infection Clearance Among Different Racial Groups

**DOI:** 10.7759/cureus.43080

**Published:** 2023-08-07

**Authors:** Rajmohan Rammohan, Sai Greeshma Magam, Melvin Joy, Dilman Natt, Achal Patel, Abhishek Tadikonda, Jiten Desai, Susan Bunting, Robert M Yost, Olawale Akande, Paul Mustacchia

**Affiliations:** 1 Gastroenterology, Nassau University Medical Center, East Meadow, USA; 2 Internal Medicine, Nassau University Medical Center, East Meadow, USA; 3 Internal Medicine, Nassau University Medical Center, East Meadow , USA; 4 Gastroenterology and Hepatology, Nassau University Medical Center, East Meadow, USA

**Keywords:** continuing health education, socio-economic factors, helicobacter pylori eradication, race-based differences, helicobacter pylori

## Abstract

Introduction

Helicobacter pylori (H. pylori) is a bacteria causing chronic stomach infections, influenced by various factors, including host traits and bacterial virulence. It uses both urease-dependent and independent mechanisms to survive acidic gastric environments. Management entails diagnosis, treatment, and eradication verification. Combining drugs is key to overcoming resistance and ensuring bacteria elimination, thus preventing recurrence and complications. H. Pylori eradication mitigates gastric cancer risk and alleviates symptoms. Racial disparities persist despite declining H. pylori and gastric cancer incidence in the United States (US). African Americans (AA) have higher gastric cancer risks than non-Hispanic Whites. Addressing these disparities is crucial to protect high-risk populations.

Methods

This study retrospectively compiled H. pylori infection data from 2009 to 2022, categorized by race. Propensity score matching balanced initial group characteristics before analysis. Chi-squared and odds ratio tests were used on the cohort, with Kaplan Meier and Log Rank methods evaluating disease clearance in ethnic groups. Data were extracted from the Sunrise Electronic Medical Record software, including patient demographics, health details, and treatment specifics. Patients aged 18-65 with H. pylori infection at Nassau University Medical Center, who followed their treatment, were selected. Data were processed using Statistical Package for the Social Sciences (SPSS) and RStudio software.

Results

The study initially included 10,040 H. pylori-diagnosed patients, with 9,288 meeting the study's criteria after attrition. Predominantly female (64.7%), the cohort was racially diverse. A longer disease clearance time was noted among Hispanics (p=0.044). Binomial logistic regression analysis identified influential factors like high school graduation rates, poverty level income, and language proficiency on disease clearance. An odds ratio analysis further emphasized language barriers (HR 0.346, p=0.043) and education status (HR 0.756, p=0.025) as primary covariates impacting disease clearance, underlining the role of socio-economic factors and language proficiency in health outcomes.

Conclusion

The study highlights racial disparities in H. pylori clearance rates, particularly among Hispanics, necessitating culturally sensitive interventions. It advocates for improved diagnostics, increased healthcare access, and social determinants of health-focused initiatives. It identifies socio-economic status and language proficiency as key factors impacting health outcomes, calling for actions to bridge these disparities. Addressing these differences can decrease healthcare inequalities and economic burden, improving overall health outcomes and reducing costs associated with H. pylori clearance.

## Introduction

Helicobacter pylori (H. pylori) is a gram-negative, helical-shaped bacterial pathogen known to cause chronic infections in the human gastric epithelium [1]. The factors contributing to H. pylori infection range from host characteristics and gastric environmental conditions to bacterial virulence factors [1]. H. pylori employs urease-dependent and independent mechanisms to endure the acidic environment of the gastric lumen [1]. Managing H. pylori involves diagnosis, treatment, and verifying eradication [2]. Successful therapy necessitates a combination of drugs to circumvent resistance [2]. Ensuring total eradication of the bacteria is critical to reducing recurrence and complications [2]. Achieving H. pylori clearance helps avert further complications, minimizes gastric cancer incidences, and alleviates patients' symptoms [3]. H. pylori and gastric cancer incidence have gradually declined in the United States [4]. However, racial disparities persist, with African American facing higher gastric cancer risks than non-Hispanic Whites [4]. H. pylori prevalence varies, ranging from 19-77% in Whites, 62-90% in Blacks, and 64-74% in Hispanics [4]. These disparities require addressing to safeguard high-risk populations against gastric cancer [4]. We study these racial disparities' effect on disease clearance in H. pylori-infected patients. The primary importance of this study lies in comprehending the racial variations among different groups in clearing H.pylori infection.

This paper was accepted for presentation at Digestive Disease Week (DDW) conference 2023 in Chicago.

## Materials and methods

Study design

Information related to H. pylori infection was retrospectively compiled from June 2009 to June 2022, with patients sorted by their race. Prior to the result analysis, the initial characteristics of each group were balanced via propensity score matching, ensuring a fair comparison. A chi-squared test and odds ratio analysis were conducted across the entire cohort. The Kaplan Meier survival analysis and Log Rank (Mantel-Cox) model were used to study disease clearance among ethnic groups. Moreover, this study aids in unearthing potential inequities in healthcare accessibility or differing health outcomes across varied racial and ethnic groups.

Data source

Data were collected retrospectively using the Sunrise Electronic Medical Record software (Allscripts, Chicago, IL). The data collected included detailed information such as age, race, gender, socio-economic status, education level, length of treatment, medication, alcohol abuse, liver disorders, upper GI bleeding, and comorbidities. This information was obtained using Current Procedural Terminology (CPT) 10 codes, including K92.2, K29.71, I86.4, I10, B96.81, 0W3P8ZZ, 0W3P7ZZ, D64.9, K70.3, K74.4, K74.5, K25.9, E08, E78.5, and K21.9.

Inclusion and exclusion criteria

The study selected all patients between the ages of 18 and 65 who were diagnosed with H. pylori infection at Nassau University Medical Center in East Meadow, New York, between 2009 and 2022. However, patients who did not maintain their follow-up appointments and those who did not complete their prescribed antibiotic therapy were omitted from the study. This exclusion likely guaranteed that any unidentified or unmeasured comorbidities that could impact patient outcomes did not skew the study results.

Data analysis

The data were processed using the Statistical Package for Social Sciences (SPSS) for Windows (from IBM SPSS Statistics, Armonk, NY) and RStudio software (from RStudio, PBC, Boston, MA). Categorical data were represented by the number of patients (n), whereas continuous data were displayed as the mean value ± the standard deviation; the p-value for the study was <0.05.

## Results

**Figure 1:**
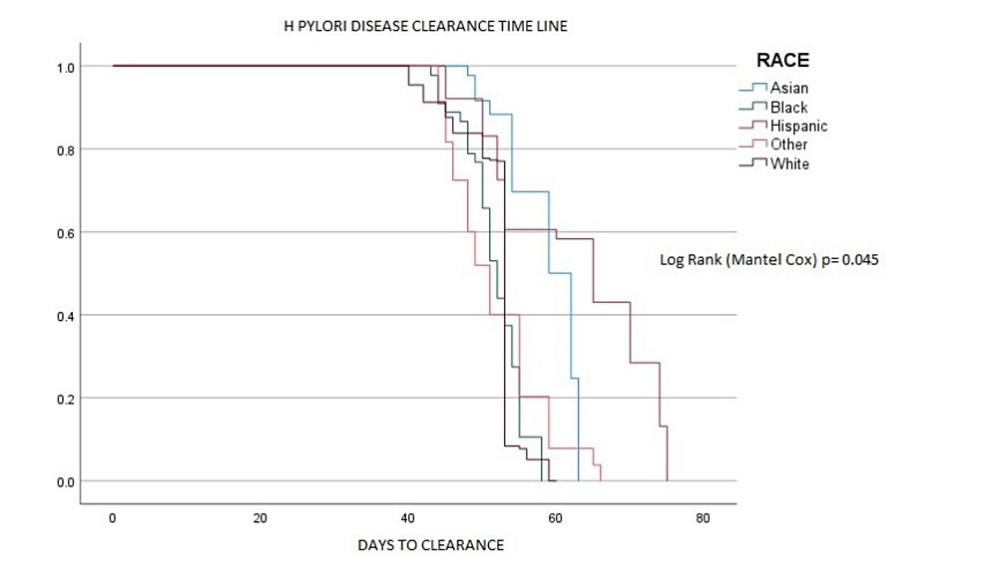
Clearance of H. pylori across different racial groups.

The research initially involved 10,040 patients diagnosed with H pylori. However, due to attrition during the follow-up period, the final group comprised 9,288 patients who fulfilled the study's criteria. The patient cohort was predominantly female, accounting for 64.7% (6,015 patients), as shown in Table 1. The racial breakdown showed a diverse patient demographic, including Hispanics (49.2% or 4,576 patients), Caucasians (18.5% or 1,725), African Americans (16.3% or 1,518), Asians (8.7% or 807), and an 'other' group (7.1% or 662). One significant observation, illustrated in Figure 1, was the longer disease clearance time among the Hispanic group compared to other racial demographics (p=0.044).

**Table 1:** Cohort characteristics values are presented as numbers (n) and mean ± standard deviation.

	N= 9288
Age in years	55.87±12.28
Sex	
Male	3273 (35.2%)
Female	6015 (65.7%)
Coronary Artery Disease (CAD)	589 (6.3%)
Anemia	795 (8.5%)
Hypertension	1516 (16.3%)
Diabetes Mellitus	1351 (14.5%)
Hyperlipidemia	1479 (15.9%)
Gastroesophageal Reflux disorder	475 (5.1%)
Heart Failure	381 (4.1%)
Chronic Kidney Disease	411 (4.4%)
Alcohol Use	674 (7.3%)

A binomial logistic regression analysis was carried out to determine influential factors affecting the disease clearance process. The results highlighted that certain variables, namely the percentage of high school graduates (p=0.012), the percentage of income below the poverty level (p=0.023), and language proficiency (p=0.011), exhibited a significant impact across all demographic groups. Subsequently, an odds ratio analysis was conducted to pinpoint primary covariates that could contribute to an extended time for disease clearance, as shown in Table 2.

**Table 2:** Racial composition of the study cohort, data are represented with a 95% confidence interval.

	Hispanic	Caucasian	African American	Asian	Other	P value
Total study cohort	4576	1725	1518	807	662	
Percentage income below the poverty level	22.3%	9.1%	20.3%	13.6%	14.4%	P= 0.023
English proficiency	5.2%	87.2%	85.6%	79.2%	64.2%	P= 0.011
Percentage graduated high school	54.2%	85.7%	83.1%	80.1 %	75.5%	P= 0.012

The principal covariates identified were language barriers (HR 0.346, p=0.043) and education status (HR 0.756, p=0.025). These factors were revealed to substantially shape the health outcomes for patients battling H pylori. This finding underscores the considerable influence of socio-economic factors and language proficiency on the health outcomes of individuals affected by this condition.

## Discussion

Racial disparities in Helicobacter pylori infection

Recent studies have shed light on the global extent of H.pylori infection, revealing a staggering number of around 4.4 billion affected individuals [4]. This implies that approximately half of the global population carries this gastric bacteria [4]. One crucial aspect to consider is that H. pylori infection is the predominant risk factor for non-cardia gastric carcinoma, a specific type of stomach cancer [5]. 

Focusing on the United States, a considerable variation can be observed in the prevalence of H. pylori infection across diverse racial and ethnic populations. It has been reported that the infection rate ranges from 18.4% to 26.2% in non-Hispanic white populations [6,7]. In stark contrast, non-white populations demonstrate a significantly higher prevalence, with rates ranging from 34.5% to a considerable 61.6% [6,7]. Taking an even closer look, specific subpopulations display remarkably high infection rates. Among these, the Alaskan Native population stands out, with an alarming prevalence of 75.0% [8]. 

Another point of interest is the comparative prevalence ratios of H. pylori infection across different racial groups. Specifically, the infection ratio for Blacks compared to non-Hispanic whites has been observed to vary from 1.3 to 5.4 [4]. Additionally, compared to non-Hispanic whites, the corresponding ratio for Hispanics falls between 1.8 and 4.4 [4]. This data underscores the disparities that exist among different racial and ethnic groups in the US when it comes to H. pylori infection.

Racial disparities in Helicobacter pylori clearance

Research indicates that African Americans show lower H. pylori clearance rates compared to other ethnic and racial groups. For example, a US study reported 74% clearance in Caucasians and 68% in Hispanics, compared to only 47% in African Americans post a 7-day treatment. The reasons behind these disparities remain unclear, possibly due to genetic factors or varied host responses to the infection [9].

Treatment failure indicated by non-serologic-based testing includes unsuccessful H. pylori eradication [10]. Around 20%-30% of US patients experience first-line therapy failure [11-13]. Eradication becomes more challenging with each failed treatment course [14]. Antibiotic resistance plays a significant role, with the global eradication rate falling to 50%-75% due to increasing resistance [15-18]. Clarithromycin and levofloxacin resistance range between 5%-25% [19-21].

Poor adherence and antibiotic resistance are two major risk factors for eradication failure [22]. Other risks include high gastric acidity, bacterial load, specific H. pylori strain insensitivity to antibiotics, and lack of awareness of local and national resistance patterns [22]. Poor adherence could result from complex regimens, adverse effects, drug cost and availability, high pill burden, or insufficient patient education. Resistance to clarithromycin and levofloxacin significantly increases treatment failure [22]. In the US, resistance rates vary based on geographical area [22]. Factors like CYP2C19, interleukin-1, and P-glycoprotein 1 (MDR1) polymorphisms indirectly decrease amoxicillin and clarithromycin efficacy [22,23]. Quitting smoking is also crucial, as it doubles the odds of treatment failure [22,23].

Possible solutions to address racial disparities in Helicobacter pylori clearance

Advancement in Screening and Identification Procedures

Infections caused by Helicobacter pylori are linked to almost half of the cases of gastric body or antrum tumors [24]. The evolution of gastric cancer is gradual, moving from preneoplastic alterations (atrophic gastritis and intestinal metaplasia) before shifting to a malignant state [25]. The key instigator for this transformative process is the Helicobacter pylori bacterial infection [25]. The removal of H. pylori infection has been proven to enhance the prognosis of peptic ulcer disease (PUD) and potentially gastric cancer [24]. Those affected by H. pylori infection undergo inflammation, which can lead to the formation of ulcers, Mucosa Associated Lymphoid Tissue lymphoma (MALT), and gastrointestinal system adenocarcinoma [26]. Despite the decreasing rates of gastric cancer in the United States, a steady increase in incidence among young Hispanic men has been noted [25]. Enhancing H. pylori and gastric cancer screening methods can lead to earlier detection and superior treatment options [27].

Currently, there are multiple diagnostic procedures for H. pylori infection, ranging from less invasive to invasive testing [28]. The most common laboratory tests include urea breath and stool antigen testing [28]. Present invasive diagnostic methods for H. pylori comprise endoscopy and biopsy-based detection, as well as rapid urease testing (RUT) and culture [28]. A 2018 study by Saumoy et al. investigated the cost-effectiveness of gastric cancer screening. The study proposed that endoscopic screening simultaneous with colorectal cancer screening, coupled with repeat surveillance every 3 years if intestinal metaplasia was detected, proved cost-effective for minority populations, including Asians, Hispanics, and non-Hispanic blacks [27]. However, this cost-effectiveness did not extend to the non-Hispanic white population within their study [27]. Early-stage screening can enable earlier detection and improved treatment options for gastric cancer [27].

Concentrated Interventions to Address Health's Social Determinants & Expanding Access to Healthcare and Treatment Options

Social determinants of health are conditions in the environments where individuals are born, live, learn, work, play, worship, and age, influencing healthcare, quality of life, and health risks over a lifetime [29]. These structures facilitate the division of the population into different socio-economic and sociopolitical groups, which are documented to affect healthcare and generate health disparities [30]. Health promotion programs, like community gardens, have been shown to assist in providing fresh groceries to socially marginalized individuals [30]. Nutritional initiatives targeting school-aged children have demonstrated effectiveness in the United Kingdom and Australia, enhancing educational performance, attendance, and punctuality [30]. Although these programs don't directly influence health outcomes, they provide additional support to parents and children and can emphasize the importance of nutrition, mental health, and literacy, thereby indirectly impacting future healthcare outcomes [30]. In a healthcare setting, addressing social determinants of health can be achieved directly by tackling existing racial and ethnic inequalities, such as language barriers [30]. Ensuring adequate patient education during hospital visits might be crucial in improving treatment compliance and follow-up visits [31]. Understanding how social determinants of health affect healthcare outcomes allows healthcare providers and policymakers to create and implement initiatives for future improvement [30].

Socio-economic status (SES) plays a vital role in patient access to healthcare [29]. In the United States, poor health outcomes are often linked to lower socio-economic status, a correlation that could be attributed to limited healthcare access among low SES adults and elderly adults [32]. Elderly adults of higher socioeconomicsocio-economic status are more likely to have access to preventative clinic appointments and receive preventative care [33]. As the global population ages, chronic diseases related to aging become a significant issue that needs to be addressed [33]. Factors facilitating improved access to healthcare, such as health insurance expansion or initiatives to bridge racial, ethnic, and financial disparities, lay a foundation for future healthcare improvement [33].

Consequences of racial disparities in Helicobacter pylori clearance

Patients with H. pylori infection have a heightened risk of developing gastric adenocarcinoma, barring tumors of the Gastro Esophageal junction. The odds ratio is 3.6 in general, but it surges to 18 in women and 9 in blacks, indicating a particularly pronounced risk in these groups [34]. If not addressed, this infection can lead to serious complications, including peptic ulcer disease and MALT lymphoma [35].

An effective response to this medical challenge is the eradication of H. pylori, which has been found to lower the rates of gastric cancer and its recurrence [36]. Eradication results in better healthcare outcomes and has profound implications for economic burden and healthcare costs. The financial stakes are enormous. Current estimates suggest that eliminating H. pylori could save the US healthcare system upwards of $14 billion [37]. This significant saving underscores the economic and health benefits of effective interventions against H. pylori, making it a vital area of focus in healthcare strategies.

The study's retrospective design may introduce biases and limit causal inferences. Using data from electronic medical records could result in incomplete or inaccurate information, potentially affecting the analysis. Although propensity score matching was employed to balance group characteristics, unmeasured confounding variables may still influence the results. Additionally, the study's focus on a single medical center may restrict the generalizability of findings to other populations or healthcare settings. The study's observational nature precludes establishing a cause-and-effect relationship between race, socioeconomic factors, language proficiency, and H. pylori clearance.

Furthermore, excluding patients who did not follow their treatment plan may introduce selection bias. Despite efforts to control for influential factors, other unexplored variables may impact disease clearance rates among different racial groups. Therefore, a cautious interpretation of the study's findings is necessary, and further research is warranted to comprehensively address racial disparities in H. pylori clearance and improve health outcomes.

## Conclusions

This study underscores the racial disparities in H. pylori clearance across diverse demographic groups. The results revealed a prolonged disease clearance time, particularly among Hispanics, emphasizing the need for culturally competent interventions. This necessitates improvements in diagnostic procedures, broader access to healthcare, and robust interventions addressing the social determinants of health. Crucially, socio-economic factors and language proficiency were found to significantly impact health outcomes in H. pylori-infected patients, warranting initiatives to bridge racial, ethnic, and financial disparities. Addressing these disparities is not only essential for reducing healthcare inequalities but also holds significant implications for the economic burden on the healthcare system. Therefore, a concerted effort toward understanding and addressing these racial disparities can improve health outcomes and reduce the overall economic cost associated with H. pylori clearance.
